# How Corporate Social Responsibility Boosts Corporate Financial and Non-financial Performance: The Moderating Role of Ethical Leadership

**DOI:** 10.3389/fpsyg.2022.871334

**Published:** 2022-05-26

**Authors:** Said Id Bouichou, Lei Wang, Salman Zulfiqar

**Affiliations:** ^1^School of Business and Management, Donghua University, Shanghai, China; ^2^Department of Management Sciences, COMSATS University Islamabad, Islamabad, Pakistan

**Keywords:** CSR, corporate image, customer satisfaction, ethical leadership, financial performance, non-financial performance

## Abstract

Corporate social responsibility has always been considered an important topic, and many studies discuss the association between corporate social responsibility (CSR) and corporate performance, but the results are still inconclusive. This study is to examine the impact of CSR on corporate performance (financial and non-financial) with the moderating impact of ethical leadership. Data is gathered from 222 companies in Morocco using a simple random sampling technique. Moreover, for measuring customer satisfaction and corporate image in the kinds of customers targeted by the CSR activities of the firms, we collected data from customers and got 209 responses. For analyzing the results of this study, structural equation modeling has been used, while for moderation, the hierarchical regression technique has been adopted. Findings revealed a significant positive association found between CSR and corporate finance as well as non-financial performance (corporate image and customer satisfaction). Ethical leadership helps in increasing the financial and non-financial performance of an organization. The findings further revealed that ethical leadership moderates the relationship between CSR and firm financial and non-financial (corporate image and customer satisfaction) performance. This study will assist management in realizing the importance and implementation of CSR practices in organizations, especially in the Moroccan context.

## Introduction

Corporate social responsibility (CSR), has been extensively studied for decades, but the results are still inconclusive and misleading (Margolis and Walsh, 2003; Mishra, 2010; Alafi et al., 2012; Galbreath et al., 2012). This study focuses on the financial and non-financial performance of the firms, to understand the importance of CSR for an organization’s success. Researchers argue for the positive association of CSR on firms’ performance (Orlitzky et al., 2003; Wu et al., 2008; Oeyono et al., 2011). At the same time, researchers discuss the negative or lack of association between the two constructs (Hatfield, 1985; Smith, 2007; Lima Crisóstomo et al., 2011). Some scholars (Rowley, 2000; Alafi et al., 2012; Galbreath et al., 2012) also questioned the approach applied in many studies to inspect the relationship between CSR and company performance. These researchers claimed that the relationship between CSR and company performance could not be consistent because this correlation can be affected by many other intervening variables neglected by previous studies. Neglecting intervening variables leads to biased results by overemphasizing the proposed relationship between CSR and corporate performance (Margolis and Walsh, 2003; Mcwilliams, 2017). The literature revealed that the actual relationship between the two constructs is much more complex than the results indicated by earlier research (Sousa and Van Dierendonck, 2017). Hence, researchers recommended that the correlation between CSR and company performance be examined through intervening variables instead of a direct association between the two (Branco, 2006). The association between the two variables would best be explained with the help of moderating or intervening variables (Orlitzky et al., 2013; Parastoo et al., 2017) which will also help in explaining this relationship and increasing the reliability of results in this domain. Accordingly, the current study extends the relationship between CSR and company performance, it will reduce the uncertainty surrounding this linkage when intervening factors are included (Galbreath et al., 2012; Castellani et al., 2016). Therefore, it can be concluded that this relationship needs further investigation (Galbreath et al., 2012; Lu et al., 2014). Accordingly, the current study extends the relationship between CSR and company performance by exploring ethical leadership as a moderator in their relationship. Much primary research in environmental management indicated customer satisfaction and corporate image as CSR outcomes (Walsh et al., 2009; Garcia et al., 2011; Mulki et al., 2011). So in this study, we have taken customer satisfaction and corporate image as determinants of corporate non-financial performance as an outcome of CSR and differentiated it from corporate financial performance. This study investigates more complex liaisons concerning CSR and company performance by comprising the moderating role of ethical leadership and argues how this leadership style impacts overall company performance (financial and non-financial). CSR is frequently linked with stakeholders’ commitment, but there is little research on leaders’ role in implementing CSR practices. CSR is considered self-serving because many companies adopt CSR activities to disguise their negligent or irresponsible behavior toward society (Hooghiemstra, 2000; Vlachos and Tsamakos, 2009). In this scenario, it is important to investigate the role of leadership by uncovering the style of leadership that can support the company’s CSR agenda and improve its financial and non-financial performance (Strand, 2011). Since ethical leaders foster integrity, fairness, accountability, and ethical behaviors (Walumbwa et al., 2011; Mayer, 2012). Therefore, we examine the role of ethical leadership in shaping CSR activities of firms.

This research aims to extend the literature on the association between CSR and company performance by including moderators, significantly impacting this relationship. Therefore, this study interrogates a new question: “Does ethical leadership moderate the relationship between CSR and company financial and non-financial performance (corporate image and customer satisfaction)?” This study responds to scholars’ call to study ethical and moral leadership perspectives in CSR. Because leaders’ moral and ethical aspects can better be related to exhibiting CSR (Siegel et al., 2006), thus, we can propose that ethical leadership offers a promising area for exploring CSR (Strand, 2011). Also, most CSR studies on its relationship with company performance have been conducted in United States and European contexts. This study is significant because the concept of CSR has not been sufficiently addressed in a study. After all, the concept of CSR has not been sufficiently addressed in the Morocco environment (Chapardar and Khanlari, 2011). Evidence also suggested that anticipation of CSR practices from Morocco firms’ stakeholders is much more than genuine CSR activities practiced by overall firms (Salehi, 2009). So, plenty of gaps exist regarding CSR practices in Morocco for this study to be conducted within (Chapardar and Khanlari, 2011; Nejati and Sasan, 2012).

## Theoretical Support and Literature Review

Behaving unethically is a serious issue because it can impact the entire company financially. This remark implies that disregarding corporate ethics can harm a firm’s performance. Firms with CSR efforts tend to develop trust-based relationships with their stakeholders when led by solid ethical leadership. Employees are motivated to seek resources (e.g., organizational support or leadership support) to protect against the actual or perceived loss of resources linked with unfortunate occurrences and complex conditions (e.g., higher work pressure, job stress, and burnout) (Ahmed et al., 2020).

We created a link between the COR perspective and significant corollaries of social support theory to support our moderation. Social support has been linked to ethical leadership as it is one of many qualities which allows the widening of the resource base beyond what an ethical leader contains within itself (Baranik et al., 2017). An ethical leader’s social interactions with their employees are considered ethical practice. Such practice shows that a leader provides an employee with actual care which develops emotions and feelings of attachment toward a person or group that is perceived to be caring (Hu et al., 2018). When the leader shows such behavior, it will positively affect the firm’s performance.

Ethical leadership is characterized by empathetic and compassionate behaviors (Ahmed et al., 2020), which can provide emotional support to employees. Companies with solid CSR are more likely to trust many stakeholders because it is a sign of good leadership (Islam et al., 2021). Necessary support provided by a leader for individuals positively impacts the company’s performance so that they show care and recognize their work efforts (Islam et al., 2020). According to research, a fundamental job of an ethical leader is to develop an organizational ethical culture. Setting an ethical example as a leader is one suggested strategy for a leader to perpetuate the desired culture (Zhu et al., 2019). In strategizing CSR activities, leaders play a crucial and leading role. This position is seen as critical for producing superior financial and non-financial results. Organizations may put CSR and performance into action with the support of ethical leaders (Lee et al., 2020). The ethical leader takes an all-encompassing approach, involving all stakeholders, initiating constructive debate, and making better judgments.

Furthermore, ethical leadership looks after all of the various stakeholders by implementing various ethical initiatives. These stakeholders, in turn, work with the company and support its initiatives, which help to improve financial performance (Sousa and Van Dierendonck, 2017). By concentrating on members’ attention to ethics and stressing priorities that guide and organize members’ actions toward reaching high levels of CSR, ethical leadership develops organizational ethical culture to increase CSR (Hu et al., 2018). Such ethical behavior fosters positive outcomes, is beneficial, and positively affects company performance (Baranik et al., 2017; Ahmed et al., 2020).

### Corporate Social Responsibility

Corporate social responsibility is an evolving concept in management literature, and much research has been directed on this topic during the last couple of decades (Nejati and Sasan, 2012). However, the application of CSR activities in the organizations started not quite so long ago (Dahlsrud, 2008), when consumers call for eco-friendly products and services (van Beurden and Gössling, 2008). CSR is considered a form of self-regulating instrument that entails how a firm integrates its societal, economic, and environmental roles by living up to the expectations of all the stakeholders (Waddock and Graves, 1997). It is considered a fundamental concept that suggests firms be responsible and conduct their business operations for the welfare of society (Freeman, 2011). Generally, CSR claims that society and business are interlinked. Much is expected from the business side as an organization, institution, and manager who ethically acted within a corporation. Though CSR is an extensively studied construct these days, there are no agreed definitions of CSR in the literature (Wood, 2010). Different researchers have defined CSR in different ways, however, all scholars agreed on one point; that corporations must equally address their economic and societal interests during their business progression (Donaldson et al., 1995). Mcwilliams and Siegel (2001) proposed CSR “actions that appear to further some social good, beyond the interests of the firms and that which is required by law. CSR is beyond obeying the law.” In another study, a more detailed explanation of CSR is stated by Garavan and McGuire (2010) as “contributing to sustainable development by working to improve the quality of life with employees, their families, the local community and stakeholders up and down the supply chain.” Carroll (1979) presented CSR in four responsibilities: economic, legal, ethical, and discretionary. Economic responsibility is aimed at producing goods or services that society requires. In terms of legal responsibility deals with laws and rules under which it functions, whereas the ethical element is concerned with societal expectations over what laws oblige. In the last, discretionary responsibility goes beyond the societal expectations mentioned above. This model differentiates organizations’ sole profit-making responsibilities from their societal and government responsibilities (Chen et al., 2012).

### Corporate Social Responsibility and Corporate Financial Performance

There is an association between CSR and company financial performance, it is believed that the literature about CSR’s impact on corporate financial performance is still inconclusive. CSR is now considered an essential part of an organization for enhancing performance. In stakeholder theory, Freeman (2011) argued that CSR positively influences firm financial performance (CFP). He stated that firms should keep all their stakeholders happy and pay attention to their concerns to ensure their maximum profit (Freeman and Phillips, 2002). Literature also suggests that employees prefer to work in socially responsible organizations and work with complete dedication to achieve those organizations’ financial goals (Backhaus and Heiner, 2002). Researchers also proposed that customers feel proud of purchasing products or services from socially responsible corporations and agree to pay extra charges for those firms engaging in CSR activities (Brammer and Millington, 2008).

Furthermore, investors favor finance in socially responsible corporations (Barnett and Salomon, 2006). These arguments propose a positive relationship between CSR and firm financial performance. Many prior studies have also shown a positive association between the two in both the short and long run (Peloza and Shang, 2010; Sheikh et al., 2010; Galbreath et al., 2012). Orlitzky et al. (2003), in their meta-analysis, analyzed 52 studies and claimed that socially responsible corporations tend to gain more profit than less socially responsible corporations. The main reason behind the positive relationship between CSR and CFP is that it gives the company a competitive advantage (Maqbool and Zameer, 2018). Corporations doing CSR activities increases competitiveness by declining organization cost and enhancing their competencies (Chang, 2016). This competitiveness in turn, enhances the economic position of the organization, and consumers are willing to pay extra toward socially responsible organizations (Castaldo et al., 2008).

Research also suggested that brand reputation, brand image, employees’ satisfaction, and customer satisfaction, boosted by CSR activities, significantly contribute to enhancing the financial performance of socially responsible organizations (Luo and Bhattacharya, 2006; Hansen and Dunford, 2011). In the same vein, Chen and Wang (2011) further argued that socially responsible firms enhance their reputation and develop a good relationship with customers, employees, stakeholders, and other people, directly enhancing their financial performance. Many researchers have confirmed the beneficial role of CSR activities for the firm. For instance, CSR is considered as a strong antecedent of customers’ willingness to pay (Pelsmacker et al., 2005) and their tendency of spreading positive word of mouth (Du et al., 2010). CSR helps create a positive perception of the firm as a whole and for the individual products or services (Bhattacharya and Sen, 2004). This loyalty results in increasing financial outcomes for the corporations, such as higher profit (Helgesen, 2010) and more customer support (Luo and Bhattacharya, 2006). Hence, we propose the subsequent hypothesis:

**H1:** CSR has a positive relationship with corporate financial performance.

### Corporate Social Responsibility and Corporate Non-financial Performance

In this study, we have distributed corporate non-financial performance into two constructs directly affected by CSR activities and cause an increase in firms’ overall performance. There is extensive literature on the correlation of CSR with corporate image and customer satisfaction. Hansen and Dunford (2011) stated that socially responsible organizations tend to enhance the corporate image and customer satisfaction level with the corporation, which increases the performance of an organization. Luo and Bhattacharya (2006) also argued in the same vein and proposed that CSR activities help firms enhance their reputation and keep a good relationship with their customers. So as the literature suggests, corporate image and customer satisfaction are considered strong determinants of corporate performance. Therefore, we have taken these terms as constructs of non-financial performance in this study.

### Corporate Social Responsibility and Corporate Image

Corporate social responsibility is one of the most significant aspects in developing a positive image for an organization, and many organizations exhibit CSR activities to show their good image in the market (Freeman, 2011). According to Fombrun (1996), corporate image is stated as “a perceptual representation of a company’s past actions and prospects that describe the firm’s overall appeal to all its key constituents when compared to other leading rivals.” He further designated four critical characteristics of corporate reputation: reliability, credibility, responsibility, and trustworthiness. There is an agreement that CSR practices are directly related to corporate image, and research also supports the fact that there is a positive association between the two constructs (Fombrun and Shanley, 1990; Lai et al., 2010; Murphy, 2011). Corporations with robust CSR activities are more likely to build trust amongst investors. Researchers also provide evidence that customers’ perception of CSR is linked with the assessment of creating an image of a particular corporation (Lai et al., 2010; Murphy, 2011). From employees’ perspective, their CSR awareness is associated with corporations’ commitment level, which improves their assessment of organizations’ image (Stawiski et al., 2010). Hansen and Dunford (2011) indicated that corporations that are socially responsible and involved in CSR actions tend to create a positive image of themselves, which enhances the performance of those corporations. CSR helps an organization build a positive image in customers’ minds, which provides a company competitive advantage and enhances employee motivation and performance (Fombrun, 1996). In another study, the researcher also suggested that corporate image results in financial and non-financial benefits for the corporations (Flatt and Kowalczyk, 2011). The same results were described by Manuel et al. (2003) and Helm (2007) stated that corporations’ having positive repute is less likely to risk and exhibit a higher level of performance and market value than corporations with a less positive image. Research also established that corporations with a positive image in customers’ minds show better profits than the corporations projecting a negative image (Roberts and Dowling, 2002). One of the reasons for doing CSR is that companies believe that it helps create a positive image, which builds trust toward customers (Rindova et al., 2005). Black and Khanna (2007) further revealed that corporations use their resources and do CSR activities to build a positive image of their corporation, expecting that it will increase performance. Therefore, we hypothesize that:

**H2a:** CSR has a positive relationship with corporate image.

### Corporate Social Responsibility and Customer Satisfaction

Customer satisfaction is considered an indication of performance for many organizations (Hinterhuber, 2003). It is necessary to satisfy customers’ needs to retain them (Kim et al., 2013). Both theories, such as institutional (Scott, 1987) and stakeholder theory (Ferrell and Ferrell, 2005), argued that a corporation’s actions are not only economical from the perspective of consumers but beyond that as the community and country members (Handelman et al., 1995). Researchers also argued that customer satisfaction is a significant factor in determining the corporate strategy in the marketing literature (Fornell et al., 2006) and for a firm’s market value and long-term success (Oh et al., 2013). Customer satisfaction determines faithfulness and excellence in customer services for a corporation and CSR activities help create customer loyalty toward the specific organization (Bharadwaj, 2009). Literature suggests that CSR is very closely linked with customer satisfaction and is a significant determinant (Galbreath, 2010; Mandhachitara, 2011). CSR can enhance customer satisfaction by enhancing corporations’ value and perceived utility (Pérez et al., 2015). Equity theory also proposes that consumers are more content with organizations that are more socially responsible toward stakeholders and customers (Pérez et al., 2015). Walsh and Bartikowski (2013) also stated that customers’ satisfaction level depends on service quality and customers’ perception of the company’s social responsibility level. Walsh and Bartikowski (2013) emphasize that CSR efforts make customers believe that they gain value in society from CSR activities performed by the organization. CSR activities help corporations’ in improving the satisfaction level of customers by appealing to and retaining those they have (Sen et al., 2009), enhancing customer loyalty (Bolton and Mattila, 2014), and improving brand image (Lee and Yoonjoung, 2009). Many of the researchers supported the fact that a strong record of CSR creates a positive image of a company in consumers’ mind and can alter their evaluation and attitude toward certain firm (Batra, 2001; Eisingerich and Seifert, 2004; Luo and Bhattacharya, 2006). CSR initiatives create key components of corporate identification that can induce customers and it make them feel a sense of connection with the corporation (Fisher and Wakefield, 1998). Research suggests that CSR adds value to the product, and customers are more prospective to drive improved apparent value and high satisfaction level with the goods produced by the socially responsible company rather than the irresponsible company. Moreover, engaging in CSR activities allows firms to improve their customer-specific knowledge and better understand their customers, which further helps firms to enhance customer satisfaction (Bhattacharya and Sen, 2004). Consequently, we can suggest the next hypothesis:

**H2b:** CSR has a positive relationship with customer satisfaction.

### Impact of Ethical Leadership as a Moderator Between Corporate Social Responsibility and Corporate Financial Performance

In the management literature, ethical leadership is stated as “the demonstration of normatively conduct specific personal actions and interpersonal relationships, and promoting such conduct to followers through two-way communication, reinforcement, and decision-making” (Brown et al., 2005, p. 19). In the social learning perspective, it is suggested that a leader influences their subordinates through modeling (Yukl and Hall, 2002) and this modeling encompasses learning from observation, imitation, and identification. This study states that one can learn from others *via* observing their behavior, and that is how leaders can influence their subordinates (Bandura, 1999). Avey et al. (2010) proposed that leaders play a significant part in developing and nourishing the ethical climate or culture within an organization. Therefore, ethical leaders are considered a source of guidance and role models for others and possess the power to enforce behaviors (Brown et al., 2005). An ethical leader is a principled individual who makes ethical decisions for society and other people (Detert et al., 2007). Ethical leadership is considered the most operative leadership style in influencing the behaviors of employees (Lam et al., 2017); that is why this leadership style is most effective in altering the behavior of employees toward implementing CSR practices in a firm.

Likewise, in the setting of this study, a leader can encourage the activities of his subordinates *via* communicating about social and ethical aspects in terms of firms’ operations (Gon and Brymer, 2011). Ethical leaders can establish clear and applicable societal responsibilities (CSR activities) and inspire employees to follow them (Thomas et al., 2004). A leader’s positive personality and strategic choice, values, and experiences influence an organization’s performance (Fahrbach, 2014). Thus, a company’s performance can be seen as reflecting ethical characteristics embodied by a leader. In this domain, empirical research has suggested that ethical leadership greatly influences an organization’s social responsibilities and, in turn, increases its performance (Carpenter and Geletkanycz, 2004). Research suggests that a firm is more likely to engage in CSR actions under solid ethical leadership because leaders implement those practices with determination and support initiatives, enhancing the firm’s overall performance (Zhu et al., 2013). Previous studies have also recommended that ethical leadership significantly motivates others to follow socially responsible behaviors (Tian and Fan, 2015). CSR is not a short-term plan, which is why it needs to be formed by strong leadership. Another research argues that strong ethical leadership takes more initiative in implementing CSR activities in the firm, which positively influences company performance (Manner, 2010). Hence, if a leader with strong ethical leadership does not participate in implementing CSR activities, then their leadership cannot positively influence the firms’ performance (Zhu et al., 2013). That is why early research supported the view that leaders tend to develop the ethical norms of the corporation (Robin and Reidenbach, 1987; Rittenburg, 1997). Researchers argued that ethical leadership produces reputational benefits such as new market opportunities, building trust, and project financing, thus generating economic benefits (Fombrun et al., 2000). Accordingly, ethical leaders enhance company reputation by implementing ethical practices and helping firms’ in adopting CSR activities which positively influence firms’ performance (Zhu et al., 2013). Thus, we can state that when leaders display strong ethical leadership then it directly effects the implementation of CSR practices for the organization which ultimately enhances performance of the firm. Based on the above arguments, we can propose the subsequent hypothesis:

**H3:** Ethical leadership moderates the relationship between CSR and company performance such that when leaders display strong ethical practices, it will positively affect the firm’s performance.

### Impact of Ethical Leadership as a Moderator Between Corporate Social Responsibility and Non-financial Performance (Corporate Image and Customer Satisfaction)

As we have discussed above, ethical leadership promotes socially responsible activities of the corporation, which further enhances the image. The influence of CSR on corporate image depends on the ethical behavior of a leader who provides the organization with an ethical climate and puts emphasis on socially responsible activities (Zhu et al., 2013). Ethical leaders tend to communicate clear ethical standards and ethical values to their subordinates and promote socially responsible practices by giving rewards for ethical behavior and punishing employees’ unethical behavior (Brown et al., 2005). Moreover, ethical leadership can maintain a corporation’s positive image and credibility by emphasizing ethical decisions and strengthening CSR practices. CSR activities depend entirely on how leaders show their authenticity in implementing CSR. Research suggested that CSR-practicing firms tend to build trustworthy solid affiliations with investors and customers. CSR is named as a device to boost a firm’s reputation, which then ultimately causes an increase in the overall performance of the firm (Bhattacharya and Sen, 2004). Prior studies have confirmed the CSR’s direct effect on the corporate image (Bhattacharya and Sen, 2004; Helm, 2007; Zhu et al., 2013) and ethical leadership moderating the relationship between them (Zhu et al., 2013). We can state that the imperceptible aspect of the corporate image is an essential source of competitive benefit, and ethical leadership provides it. Researchers claim that CSR is certainly linked with the corporation’s positive image building when leaders display strong ethical behaviors and implement CSR practices with determination, supported by solid actions which then ultimately enhance the reputation or image of the firm (Batra, 2001; Brown et al., 2005; Zhu et al., 2013). Researchers also suggested that CSR advantages provide reputational rewards for the corporation, such as trust-building, exploring new opportunities for the firm, and getting financing for projects (Batra, 2001; Brown et al., 2005; Zhu et al., 2013). Many types of research have indicated that CSR activities help firms gain a reputation that then generates a positive image of the brand (Smith and Higgins, 2000; Brickley et al., 2002). Therefore, we propose that robust ethical leadership has an unintended effect on creating a positive image of the corporation through implementing CSR.

Many corporations are using customer satisfaction to determine the performance of a firm (Hinterhuber, 2003) and CSR activities help firms satisfy customers. CSR directly influences customer satisfaction by developing a positive image of the corporation and themselves as they believe that they are doing something good for society (Pérez et al., 2015). The research also suggests that ethical leadership help in establishing CSR activities constantly in an organization by communicating clear ethical guidelines and standards to their followers and promoting socially responsible practices (Brown et al., 2005). These constant efforts then help an organization build a positive attitude toward certain companies and make customers believe that they gain value from those companies and improves their satisfaction level (Sen et al., 2009). It has been argued that ethical leaders tend to create trustworthy relations with customers by exhibiting CSR practices which result in customer satisfaction (Brown et al., 2005; Zhu et al., 2013). Many studies supported that CSR activities help create a positive image of a firm in the mind of consumers and change consumers’ attitudes toward that firm (Eisingerich and Seifert, 2004; Luo and Bhattacharya, 2006). Another study states that CSR positively affects customer satisfaction (Alafi et al., 2012). Ethical leadership emphasizes taking socially responsible decisions for the corporation, which adds positive value to the firm’s image and customers are more prospective to drive a high satisfaction level with a socially responsible firm rather than an irresponsible firm.

Moreover, these activities improve firms’ customer-specific knowledge and allow the firms to understand customers better, resulting in customer satisfaction (Luo and Bhattacharya, 2006). Therefore, it is evident that ethical leadership helps corporations exhibit CSR, positively influencing customer satisfaction with that corporation. Therefore, we recommend the following hypothesis:

**H4:** Ethical leadership moderates the relationship between CSR and non-financial performance (corporate image and customer satisfaction) in such a way that when leaders display ethical practices, it will positively affect the non-financial performance of the firm.

## Methodology

### Sample and Data Collection

The current study is based in Morocco, and as no such database like Fortune’s Most Admired Companies or Kinder Lindenberg and Domini (KLD) exists for the country, the best approach is to use a survey method to collect data for this study mainly because no secondary data was available. We developed a self-administrative, 5-point Likert scale based on extensive literature for data collection. For ensuring the secrecy of the respondents and companies, their approval was taken before conducting this study. Furthermore, detail of the complete questionnaire was shared with respondents as well. The feedback of 15 executives has been considered to evaluate the content validity of our instrument. After making minor changes suggested by executives, the corrected and final version of the questionnaire was prepared. The study undergoes the guidelines suggested by Spector and Brannick (1995) to reduce biases and does not form any preferred answers in our instruments. Also, we randomly ordered dependent and independent variables, and reverse coding is used; also, attention is paid to the questionnaire wording, and the questionnaire is kept as short as possible. Data is collected from managers of listed companies in Morocco as they are the ones who are directly involved in making organizational decisions and management of organizational affairs. Using simple random sampling, we distributed a total of 750 questionnaires among managers of listed companies. In return, we got 200 and 57 responses from respondents, of which 35 responses were disqualified due to incomplete records. After removing unusable responses, 222 responses were used for analysis giving a response rate of 29.6% for the study. To measure corporate image and customer satisfaction, we distributed the questionnaires to the general public to know their intent toward firms practicing CSR activities. Therefore, we distributed 500 questionnaires to customers, and we got only 209 responses back, which gives us a 41% response rate for customers. The response rate also illustrates that the impression of CSR is considered unimportant for Morocco, which is why we conducted this study in Morocco. As Welford (2004) stated, the response rate suggests the importance of certain concepts perceived in any country, and the low response rate of CSR suggests that it is perceived as not so necessary for that country. That is why a study on CSR in developed countries yields a high response rate because they give more importance to CSR than developing countries. However, in their research, Galbreath and Galvin (2008) described that as low as 10% response rate is satisfactory for countries that undergo severe deficiency of survey and research work on CSR. Thus, based on that rationale, a response rate of 29% for managers and 41% for customers is more than enough for an emerging economy like Morocco where the concept of CSR has not been adequately addressed.

### Measures

#### Corporate Social Responsibility

There is no universal and unified method to conceptualize CSR, as researchers argued (Montiel, 2008; Galbreath and Shum, 2012). CSR was measured using a 13-item scale developed by Singhapakdi et al. (1996) to measure top managers’ perception of CSR. We used the Likert scale (ranging from 1 = disagree to 5 = agree) for all the questionnaire items. The sample of this item includes: (a) “Social responsibility and profitability can be compatible,” (b) “Being ethical and socially responsible is the most important thing a firm can do,” and (c) “Business has a social responsibility beyond making a profit.” This instrument has been extensively used in many studies and validated in the management literature (Etheredge, 1999; Shafer et al., 2007). The reliability of this scale is 0.71 (Singhapakdi et al., 1996).

#### Ethical Leadership

For measuring the ethical leadership of managers, we used a 5-item scale from Brown et al. (2005) to measure ethical leadership, which is most according to our study. The sample of the items are: (a) “I discuss business ethics or values with employees,” (b) “I set an example of how to do things the right way in terms of ethics,” and (c) “I ethically conduct my personal life.” The scale’s reliability is 0.80 (Brown et al., 2005).

#### Corporate Image

The corporate image instrument comprised 5-items and was taken from Bayol et al. (2000). The sample of the items are: (a) “It can be trusted in what it says and does; (b) It is stable and firmly established”; and (c) “It has a social contribution to the society.”

#### Customer Satisfaction

The customer satisfaction scale consisted of seven items adapted from Galbreath and Shum (2012). The sample of the items include: (a) “Compared to competitors, our customers find that our products/services are much better”; (b) “Our customers are delighted with the product/services we offer”; and (c) “Our customers are delighted with the value for the price of our products/services.” The scale’s reliability is 0.87 (Galbreath et al., 2012).

#### Financial Performance

In the end, the scale of financial performance was measured using the balanced scorecard approach (BSC) established by Norton and Kaplan (1992). The BSC measures overall company performance in four dimensions, and financial performance is one of its dimensions. This study considers assets, return on investment (ROI), sales, equity, and profit margin as constructs of financial performance. We adopted this approach as it is considered the most acceptable approach for measuring financial performance (Saeidi et al., 2015).

The company’s size is considered a control variable, and we measured it by asking one question. This question was about the number of working employees in the corporation. However, the result is quite insignificant and in line with the previous studies (Webb, 2004; Galbreath and Shum, 2012). Prior studies also suggest that control variables have no significant effect on CSR. For analyzing the data, descriptive and inferential statistics have been adopted. Cronbach alpha has been used for reliability analysis for each variable.

Moreover, structural equation modeling (SEM) is used for analysis. Also, variance inflation factor (VIF) values are used to check multicollinearity issues in our model, and all VIF values were found to be within an acceptable range. The analysis for reliability, correlation, and descriptive analysis has been done with the help of SPSS 22. SEM has been done through AMOS 22. For moderation, the hierarchical regression technique has been used as it has been considered one of the best techniques for analyzing the interaction between variables (Cohen, 1983).

## Data Analysis

### Demographic Information of the Sample

Data is gathered from the managers of listed companies in Morocco. Table 1 shows the demographic information of the sample. Table values indicated that most respondents (54%) have 5–9 years of experience. At the same time, the second majority of respondents were found to have 1–4 years of experience. Whereas more than 18 respondents had less than 1 year of experience, and 24 respondents had more than 9 years experience, respectively. In the case of manager positions, most of the respondents (68%) were middle-level managers, whereas only 16% were top-level managers. At the same time, 14% of our respondents were first-line managers. In the case of the number of employees, our results exposed that most corporations (5%) have 100–500 employees while 36 companies have less than 100 employees. At the same time, only 19 organizations had more than 500 employees working for them. Moreover, in the case of customers, we got 209 responses (41%) from existing or potential customers.

**TABLE 1:** Demographics of the sample.

Demographics		Percentage	Frequency
Experience (years)	Less than 1	8	18
	1 to 4	26	58
	5 to 9	54	122
	9 and over	10	24
Position	Top managers	16	37
	Middle managers	68	153
	First-line managers	14	32
Number of employees	Less than 100	16	36
	100 to 500	75	167
	500 and more	8	19
Customers	Existing and non-existing	41	209

### Common Method Biasness

As we gathered data from a single source using questionnaires, we need to examine the common method variance for our study. Therefore, the Herman test has been preferred for this purpose. The test results show that a single factor explains 43.5% variance within the threshold value and under the acceptance level. So, we can conclude that our data do not have an issue of Common Method Bias (CMB). Moreover, we also used Kaiser–Meyer–Olkin (KMO) and Bartlett’s test to measure the adequacy of our data. The value of KMO for our data was 0.805, which is beyond the threshold value (Hair et al., 1998). Hence, we can show that our data is adequate and run a confirmatory factor analysis (CFA) based on our results.

### Analysis and Results

Table 2 values of mean, correlation, and standard deviation are given.

**TABLE 2:** Descriptive and correlation analysis of variables.

Mean	SD		CR	AVE	MSV	MaxR (H)	EL	CS	CSR	FP	CI
**3.95**	**0.54**	**EL**	0.800	0.574	0.307	0.820	**0.758**				
**4.28**	**0.53**	**CS**	0.849	0.652	0.403	0.849	0.497	**0.807**			
**3.89**	**0.55**	**CSR**	0.866	0.683	0.403	0.880	0.554	0.635	**0.827**		
**3.74**	**0.69**	**FP**	0.759	0.510	0.304	0.770	0.033	0.017	0.022	**0.715**	
**4.12**	**0.59**	**CI**	0.761	0.516	0.288	0.767	0.380	0.455	0.537	0.046	**0.718**

*Bold indicates the square root of AVE.*

According to Table 3, measured levels of the goodness of fit index (GFI), root mean square (RMR), and comparative fit index (CFI) are shown, the values for GFI and CFI should be more than 0.9 and in the case of RMR, the value should be less than 0.05 and as our results showed, all values lie in the acceptable range.

**TABLE 3:** Confirmatory factor analysis results.

Construct	Mean	SD	Internal consistency	Cronbach alpha	CFI	GFI	RMR
CSR	3.89	0.55	0.82	0.827	0.94	0.90	0.03
EL	3.95	0.54	0.76	0.758	0.91	0.91	0.01
CS	4.28	0.53	0.81	0.807	0.92	0.94	0.03
CI	4.12	0.59	0.74	0.718	0.96	0.93	0.02
FP	3.74	0.69	0.73	0.715	0.93	0.95	0.01

Exploratory Factor Analysis (EFA) and CFA is adopted to evaluate the factor structure. EFA is used to analyze factor structure, whereas CFA is used for confirmation analysis. Therefore, CFA has been used to estimate the validity of our constructs used in the study (Hair et al., 1998). According to Uma Sekaran (2003), the Cronbach alpha of less than 0.7 value is termed weak, while above 0.7 value is considered strong and reliable. Cronbach alpha values for this study are also given in Table 3. Also, internal consistency between variables should be close to 1 suggests good reliability (Uma Sekaran, 2003). In the study, values showed consistent reliability for all the variables and were within the range of the benchmark value of 0.7. Moreover, the factor loadings also showed consistent results and revealed that the convergent validity is good for all the items.

According to Rowley (2000) and Galbreath and Shum (2012), SEM is the most suitable approach to hypothesis testing for CSR research. Iacobucci et al. (2007) also claimed that SEM is a better statistical and theoretical analysis approach that simultaneously explains the causative association between several constructs (independent and dependent variables). Researchers also suggested that SEM showed improved results than regression since it can diminish the biasedness involved by considering measurement errors (Urbach and Ahlemann, 2010). Therefore, we used SEM for the testing of hypotheses in this study.

The first hypothesis for the relationship showed that the association between CSR and corporate financial performance is significant (β = 0.457, *t* = 7.030), suggesting that H1 is accepted. Whereas in the case of the second hypothesis, the results of the association between CSR and corporate financial performance (corporate image and customer satisfaction) are shown. In relation with the corporate image, CSR also shows significant (β = 0.432, *t* = 6.496) results. While in the case of customer satisfaction, results are also significant (β = 568, *t* = 8.564). So our hypotheses H1 and H2 (H2a and H2b) are accepted.

For determining the moderator relationship, we will use regression results of chain moderation for our analysis. The results of our hypotheses H3 and H4 are shown in Tables 4, 5.

**TABLE 4:** Main effect variables.

	Estimate	SE	CR	*p*-Value
FP ← CSR	0.457	0.056	7.030	***
CS ← CSR	0.568	0.66	8.564	***
CI ← CSR	0.432	0.067	6.494	***

***p < 0.001.

**TABLE 5:** Hierarchical regression results of moderator.

Construct	Model 1 Financial performance	Model 2 Corporate image	Model 3 Customer satisfaction
CSR	0.415***	0.465***	0.408***
Ethical leadership	0.492***	0.426**	0.483***
CSR × ethical leadership	0.390***	0.412**	0.539*

^***^p < 0.001, ^**^p < 0.01, *p < 0.05.

The findings of the moderation for H3 shows that under ethical leadership, the financial performance (β = 0.390, *p* < 0.001) of the corporation is quite significant, which suggests that under strong ethical leadership, the corporation is likely to be more involved in CSR practices which in turn increases the financial performance of the corporation as shown in Figure 3.

**FIGURE 1:**
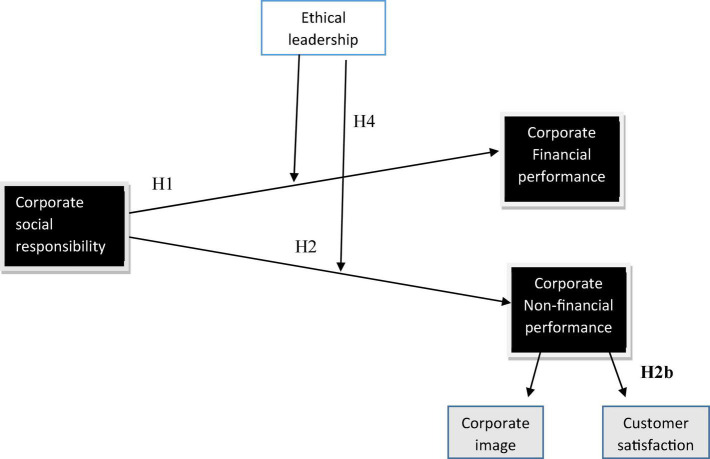
Theoreticalframework.

**FIGURE 2:**
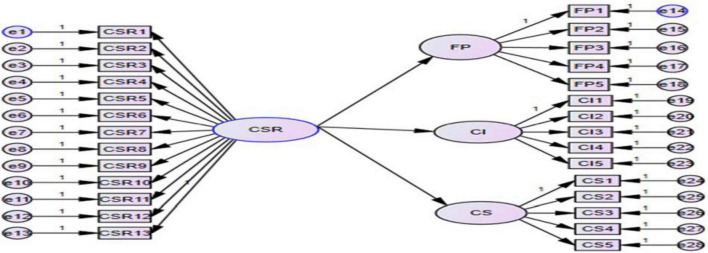
Structural model.

**FIGURE 3:**
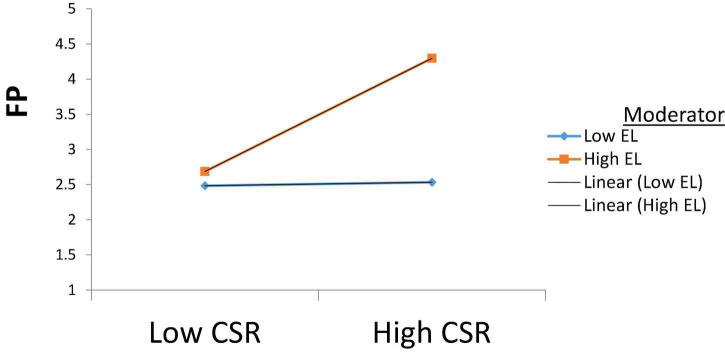
Moderating role of Ethical leadership on CSR and FP.

**FIGURE 4:**
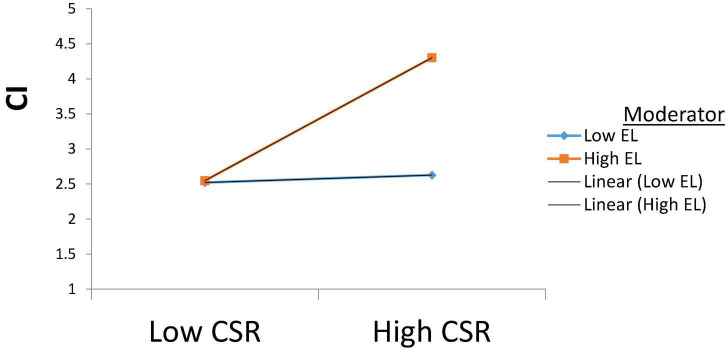
Moderating role of Ethical leadership on CSR and CI.

While in the case of H4a, the moderating results of ethical leadership show significant results (β = 0.412, *p* < 0.001) with the corporate image, which suggests that strong ethical leadership helps a firm build a positive image of a firm by executing consistent CSR practices. This relationship is illustrated in Figure 4.

**FIGURE 5:**
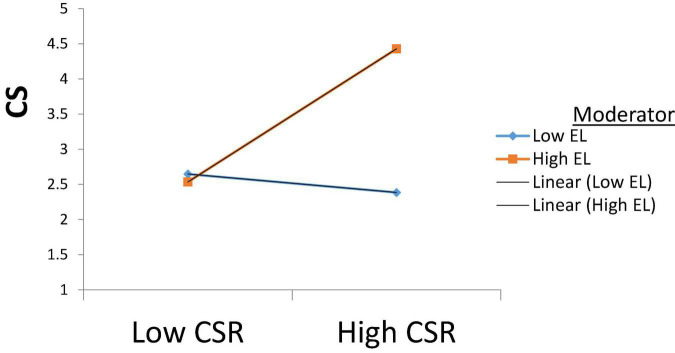
Moderating role of Ethical leadership on CSR and CS.

Further, results for customer satisfaction also show significant results (β = 0.539, *p* < 0.05) with the moderator, which suggests that ethical leaders by implementing CSR practices in a firm help satisfy customers. This relation is shown in Figure 5. Thus we can state that all our hypotheses are accepted.

## Discussion

The relationship between CSR and firm financial performance has been debated previously in different cultures and settings. Many studies suggest a positive association between CSR and company performance (Maignan et al., 1999; Oeyono et al., 2011; Orlitzky et al., 2013). Some studies argued for negative or no relationship between the constructs (Hatfield, 1985; Smith, 2007; Lima Crisóstomo et al., 2011). Only a few researchers followed the contingency approach in measuring this association (Margolis and Walsh, 2003; Luo and Bhattacharya, 2006; Galbreath et al., 2012). Previous researchers ignored the impact of moderating variables, particularly in developing countries like Morocco. Branco (2006) argued that intervening variables should be investigated to explore the actual effects of CSR on company performance. Thus based on the rationale and statements made by the researchers, this study includes ethical leadership as a moderator to show the influence of this leadership style on implying CSR activities in the firms and how it affects firms’ performance. The current study is an addition to the literature in the context of developing economies because CSR is not given that much importance and there are very few studies on CSR, especially in Morocco (Chapardar and Khanlari, 2011). The findings of this study demonstrated that CSR is positively related to company financial and non-financial performance (corporate image and customer satisfaction). These findings are consistent with the prior researches (Belanche Gracia et al., 2015; Hasan et al., 2018). The study determines the impression that CSR positively influences the corporation’s performance. This study also affirms that any corporation involved in CSR activities builds its positive image in the mind of customers, which then enhances customer satisfaction with the firm. These results reaffirm the findings of previous studies (Navickas and Kontautiene, 2011; Maruf, 2013). All these factors increase customers’ confidence, trust, and loyalty level with the firm, which are considered the leading indicators of enhancing the firm’s economic performance (Saeidi et al., 2015). Therefore based on our results, we can state that the firms that devote their assets to CSR activities gain more profits ultimately because of having a better reputation than those firms that invest not as much in socially responsible activities (Wang et al., 2016). Consumers are willing to pay for those companies involved in socially responsible activities, which also enhances the company’s performance. Flatt and Kowalczyk (2011) have also suggested that CSR activities help create a positive corporate image of an organization, which is very important for firms’ monetary and non-monetary benefits.

The inconsistent results concerning company performance previously also indicate a need to understand better how it happens. In this regard, the results of ethical leadership as a moderator also suggest that it moderates the association between CSR and firm financial and non-financial performance. CSR has a solid indirect effect on increasing the performance of a corporation when leaders show robust ethical leadership practices; its effect is diminished when leaders display weak ethical practices. Under strong ethical leadership, a corporation is well engaged in CSR activities and follows the instructions given by an ethical leader, which helps a firm enhance its overall performance (Zhu et al., 2013). This leadership approach explains the sustainability and legitimacy in achieving success for a business, and also it serves as a tool for implementing responsible behaviors in an organization and toward society as well (Sama and Shoaf, 2008). However, companies adopt CSR to get benefits from society. CSR is now observed as a business strategy or asset for a corporation to differentiate its products and services. Moreover, this differentiation allows the firms to demand premium prices for their products or services. Mcwilliams and Siegel (2001) proposed that “CSR may be a popular means of achieving differentiation because it allows ethical leaders to simultaneously satisfy personal interests and achieve product differentiation,” which positively impacts the firm’s performance.

Finally, it can be stated that CSR has a constructive impact on firm financial and non-financial performance. By becoming involved in CSR, companies make their image better and help them satisfy their customers, and ethical leadership help in creating a positive relationship between these. CSR is not correctly implemented and followed in Moroccan companies, and there is a need to enhance CSR activities to gain better results. However, the findings of this study approve the positive impact of CSR in developing countries like Morocco as it is perceived in developed countries and all over the world (Galbreath and Shum, 2012).

## Practical Implication

Corporate social responsibility is considered an important aspect to obtain monetary objectives for businesses. Many researchers have argued the direct association of CSR with company performance. Moreover, most of these researches discuss the positive influence of CSR on corporate performance. At the same time, few of them disregarded this association. Some researchers have criticized the approaches used to measure the relationship and they questioned the mechanism’s reliability because of the possibility of mediation and moderation involved in the relationship.

Moreover, most of the literature is based on developed economies such as the United States and European contexts. Hence, a study on Morocco can help better understand the CSR concept for developing economies. This study is significant because CSR has not been adequately addressed in a developing environment (Chapardar and Khanlari, 2011). Studies also advised that anticipation of CSR practices from Moroccan stakeholders of firms are more genuine CSR activities practiced by overall firms (Salehi, 2009). Thus, there are plenty of gaps for this study to be conducted in terms of CSR practices in Morocco (Chapardar and Khanlari, 2011; Nejati and Sasan, 2012). Therefore, we chose Morocco as it has not been widely discussed in CSR. Also, this study can generalize the results to other such countries where CSR is not actively practiced (Figures 1, 2). So, this study provides value to the literature in terms of theory and practice as it discussed the role of CSR for less-developed countries like Morocco and thus can be generalized for these nations. Based on these logical claims and rationale, this study is conducted to overcome this gap and examined the relationship between CSR and company performance with the moderating role of ethical leadership in the Moroccan context. This study concludes that CSR significantly improves the financial performance of the corporation. This study also settles that CSR helps an organization build its positive image and by being involved in socially responsible activities, it helps enhance customer satisfaction level with the corporation, and these constructs are strong indicators of performance.

Furthermore, this study concludes that a firm behaves more socially responsible under strong ethical leadership, which enhances corporate image and customers’ level of satisfaction with the corporation. Ethical leadership leads an organization to follow CSR practices consistent with the determination which help an organization achieve better financial performance. So, the present study extends the literature on the affiliation between CSR and corporate financial and non-financial performance in general and from the Moroccan perspective in particular by examining the moderating effect of ethical leadership.

## Limitations and Future Research

This study has some limitations and also directions for future research. This study investigating the relationship between CSR and corporate performance (financial and non-financial) only focused on the positive aspects of CSR activities and disregarded the potential risks and costs associated with implementing CSR practices that might lessen the performance of a company. Since we obtained data from managers only, there is a possibility of bias involved. For future studies, it could be useful to obtain data from multiple stakeholder groups such as employees, suppliers, or competitors. Also, this study is limited to one geographical region in Morocco, which may limit the globalization of our results. Future studies could investigate a similar type of study in some other regions and countries to enhance external validity.

Moreover, future research can contain samples from different countries or regions to recognize comparatively general settings across different boundaries (Liden, 2012). This study is focused on Moroccan listed companies, and only cross-sectional data was used. Data can also be collected for extended periods to add credibility to results. Future research can adopt panel data, and the sample size can also be increased. Moreover, researchers can explore the relationship between CSR and company performance by using other necessary contingencies of personality traits of an ethical leader and different orientations of it. In the end, CSR can also be studied in some other contexts, like hurdles that restrain corporations from practicing CSR in developing economies can also be studied in future research.

## Data Availability Statement

The original contributions presented in the study are included in the article/supplementary material, further inquiries can be directed to the corresponding author.

## Author Contributions

SB: conceptualization, methodology, and software. LW: supervision, validation, review, and editing. SZ: visualization and investigation. All authors contributed to the article and approved the submitted version.

## Conflict of Interest

The authors declare that the research was conducted in the absence of any commercial or financial relationships that could be construed as a potential conflict of interest.

## Publisher’s Note

All claims expressed in this article are solely those of the authors and do not necessarily represent those of their affiliated organizations, or those of the publisher, the editors and the reviewers. Any product that may be evaluated in this article, or claim that may be made by its manufacturer, is not guaranteed or endorsed by the publisher.
